# Stability of the Halide Double Perovskite Cs_2_AgInBr_6_

**DOI:** 10.1021/acs.jpclett.3c00303

**Published:** 2023-03-21

**Authors:** Yukun Liu, Iver J. Cleveland, Minh N. Tran, Eray S. Aydil

**Affiliations:** Department of Chemical and Biomolecular Engineering, Tandon School of Engineering, New York University, Brooklyn, New York 11201, United States

## Abstract

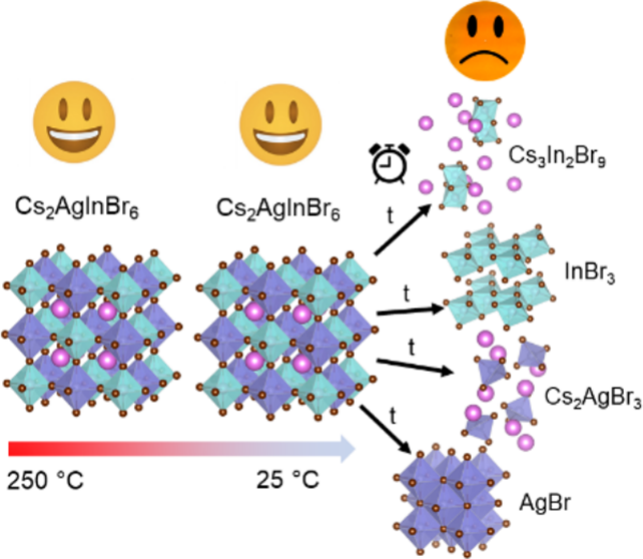

Cs_2_AgInBr_6_ is among the lead-free halide perovskites of interest, predicted by first-principles calculations to be stable with a direct band gap, but there has been only one report of its synthesis. Herein we report the formation of Cs_2_AgInBr_6_ thin films through thermal evaporation of CsBr, AgBr, and InBr_3_ and subsequent annealing between 130 °C and 250 °C. Cs_2_AgInBr_6_ appears stable in this temperature range. However, Cs_2_AgInBr_6_ thin films are thermodynamically unstable at room temperature, remaining cubic only long enough to be characterized but not long enough to be useful for practical devices. Cs_2_AgInBr_6_ decomposed into Cs_2_AgBr_3_, Cs_3_In_2_Br_9_, AgBr, and InBr_3_ upon cooling from 130 °C to 250 °C to room temperature. This conclusion did not depend on illumination, film thickness, annealing environment, or details of the film formation, pointing to an intrinsic thermodynamic instability of the material. Optical absorption measurements may be interpreted as Cs_2_AgInBr_6_ having a direct band gap of 1.57 ± 0.1 eV.

Inorganic metal halide double perovskites with chemical formula Cs_2_BB′X_6_, where B is a monovalent cation (e.g., Ag^+^, Cu^+^, Na^+^), B′ is a trivalent cation (e.g., Bi^3+^, Sb^3+^, In^3+^), and X is a halide (e.g., Cl^–^, Br^–^, I^–^), are explored as lead-free alternatives to the widely studied CsPbX_3_ in various optoelectronic applications.^1−4^ Of the various combinations of B, B′, and X, Cs_2_AgInBr_6_ is one of the least experimentally investigated double perovskites^5^ despite being predicted to be thermodynamically stable using density functional theory (DFT) calculations by many studies.^6−13^ Dai et al. used the hybrid HSE06 functional and calculated that Cs_2_AgInBr_6_ is a direct bandgap material with a bandgap (*E*_g_) of 1.25 eV.^6^ Xu et al.^7^ used the HSE06 functional but also considered spin–orbit coupling and predicted a direct bandgap of 1.33 eV and a cubic (*Fm*3̅*m*) lattice parameter *a* = 11.20 Å, close to the values *E*_g_ = 1.50 eV and *a* = 11.16 Å calculated by Zhao et al.^8^^8^ and *E*_g_ = 1.50 eV and *a* = 11.20 Å by Li et al.^13^. The cubic Cs_2_AgInBr_6_ structure can be visualized as a network of corner-sharing AgBr_6_ and InBr_6_ octahedra that alternate along the three-cube axis with Cs^+^ ions in the cavity formed by the cube octahedra. The first synthesis of Cs_2_AgInBr_6_ was achieved using the mechanochemical approach by Breternitz et al.,^5^ who reported a direct bandgap of 2.36 eV measured using diffuse reflectance, significantly higher than the values predicted from the DFT calculations (1.25–1.50 eV),^6−8^ and found that the material is unstable under illumination. The reflectance, however, started to rise gradually at around 1.7 eV, closer to the values calculated from DFT. The authors speculated that this might be due to parity-forbidden transitions, as predicted by Meng et al.^14^ Breternitz et al. reported a cubic lattice parameter of 11.0 Å.^5^ To our knowledge, this is the only reported synthesis of Cs_2_AgInBr_6_.

We co-evaporated CsBr, AgBr, and InBr_3_ in a 2:1:1 stoichiometric ratio onto glass substrates in a high-vacuum physical vapor deposition (thermal evaporation) chamber^15−17^ and formed Cs_2_AgInBr_6_ thin films after post-deposition annealing. The films were characterized either as deposited or after annealing (in the air or N_2_-filled glovebox) using a battery of methods, including *in situ* X-ray diffraction (XRD) while annealing in air. The Experimental Methods section contains the deposition, annealing, and characterization details. We show here that the cubic Cs_2_AgInBr_6_ films are formed after annealing at temperatures above 100 °C but are stable at room temperature for only 1–2 h, just long enough to be characterized but not long enough to be useful in any applications.

Figure 1a shows XRD patterns from an as-deposited film at room temperature and at 130 °C, 325 °C, and 350 °C. XRD from the as-deposited film at room temperature exhibits diffraction peaks that can be identified as those from Cs_3_In_2_Br_9_ (2θ = 23.1° and 2θ = 26.6°),^18^ Cs_2_AgBr_3_ (2θ = 28.9° and 2θ = 38.1°),^19^ AgBr (2θ = 44.5°),^20^ and InBr_3_ (2θ = 32.8°),^21^ indicating only partial reaction between CsBr, AgBr, and InBr_3_ during the deposition to form the ternary phases with no evidence of Cs_2_AgInBr_6_ formation. Figure 1a also shows the calculated XRD patterns for *a* = 11.00 Å and *a* = 11.20 Å (the two values reported in the literature)^5,13^ the XRD patterns for Cs_3_In_2_Br_9_,^18^ CsAgBr_2_,^22^ and Cs_2_AgBr_3_^19^ as possible impurity phases, and those of the unreacted InBr_3_,^21^ CsBr,^23^ and AgBr^20^ precursors. When heated to 130 °C, the same film exhibits only XRD peaks that can be identified with Cs_2_AgInBr_6_, confirming its formation via the overall reaction 2CsBr + AgBr + InBr_3_ → Cs_2_AgInBr_6_. We compare the XRD pattern recorded at 130 °C to the simulated cubic Cs_2_AgInBr_6_ XRD pattern using *a* = 11.00 Å and *a* = 11.20 Å, the two previously reported lattice parameters. Our film’s XRD pattern at 130 °C shows a cubic lattice parameter of 11.04 Å, close to but slightly higher than 11.00 Å, the previous experimentally measured value at room temperature.^5^ As the film is heated to 325 °C, the lattice parameter, *a*, expands to 11.16 Å, and all peaks shift to lower 2θ values. The XRD pattern at 325 °C shows an additional peak at 2θ = 27.1°, which may be from CsAgBr_2_ (600) or InBr_3_ (130), planes indicating the possible decomposition of Cs_2_AgInBr_6_ at this temperature. Cs_2_AgInBr_6_ peaks persist, and the XRD pattern becomes textured along the (400) direction at 350 °C. However, when this film is cooled to room temperature, all Cs_2_AgInBr_6_ XRD peaks disappear and are replaced by diffractions that may be identified as coming from Cs_2_AgBr_3_, Cs_3_In_2_Br_9_, AgBr, and InBr_3_. This progression suggests that Cs_2_AgInBr_6_ may form but is unstable.

**Figure 1:**
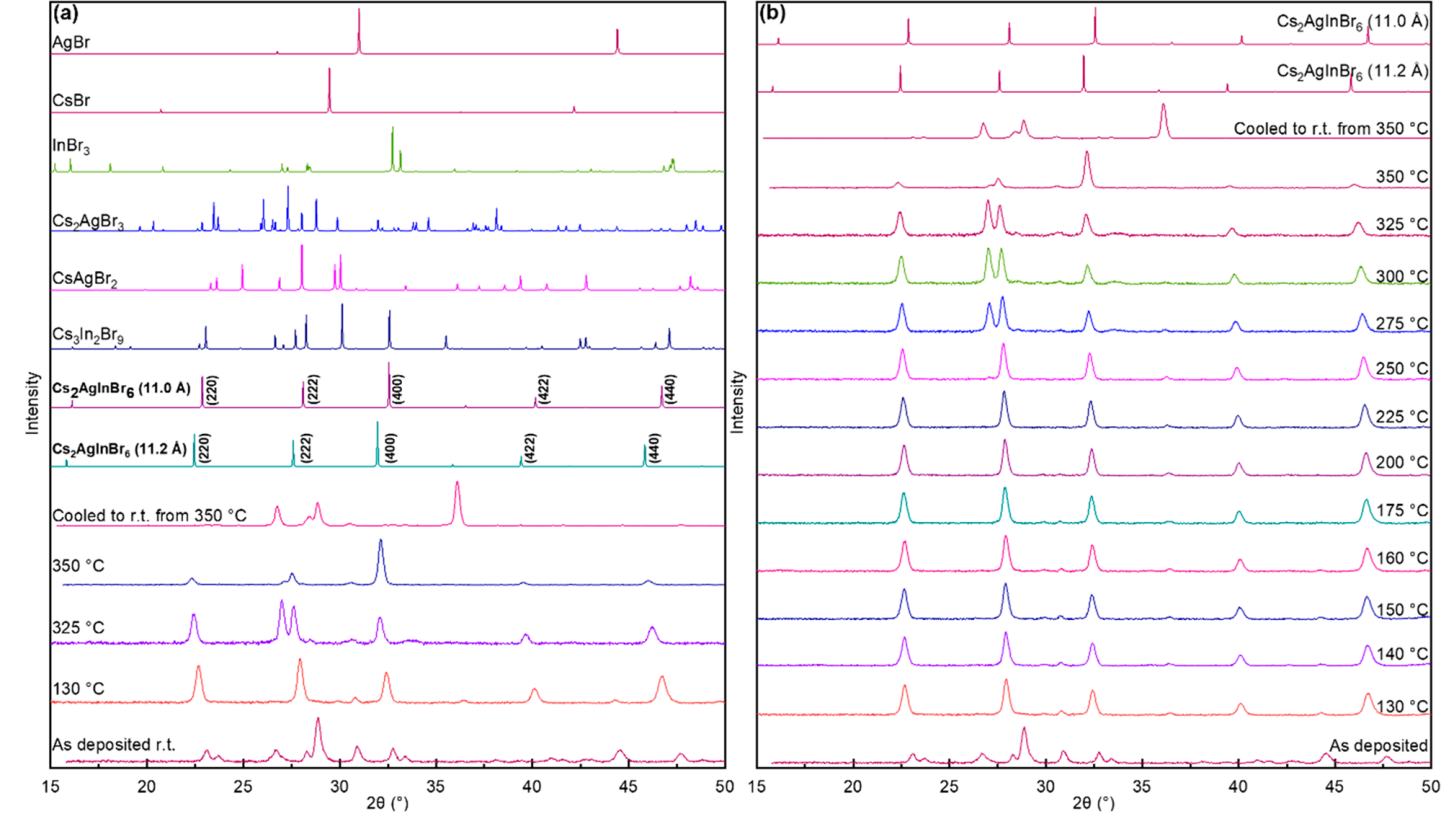
(a) XRD patterns (from bottom to top) of the as-deposited film at room temperature, 130 °C, 325 °C, 350 °C, and after cooling to room temperature. Simulated XRD patterns for Cs_2_AgInBr_6_ with *a* = 11.2 Å and *a* = 11.0 Å and potential impurity phases and precursors are also shown for comparison. (b) *In Situ* XRD patterns (from bottom to top) of the as-deposited film as it is heated at room temperature (labeled as “As-deposited”), 130 °C, 140 °C, 150 °C, 160 °C, 175 °C, 200 °C, 225 °C, 250 °C, 275 °C, 300 °C, 325 °C, and 350 °C and after cooling to room temperature. Calculated XRD patterns for Cs_2_AgInBr_6_ with *a* = 11.2 Å and *a* = 11.0 Å are shown in (b) for comparison. The film thickness was 510 nm.

Figure 1b shows the XRD patterns collected from the as-deposited film as it was heated from room temperature to 350 °C while maintaining the temperature at intermediate values. The XRD pattern recorded at 130 °C already shows the peaks at 2θ = 22.6°, 28.0°, 32.4°, 40.1°, and 46.8° corresponding to the (220), (222), (400), (422), and (440) planes of Cs_2_AgInBr_6_, respectively. The lattice parameter calculated from these peaks at 130 °C is 11.04 ± 0.05 Å, between the experimentally reported value at room temperature, 11.0 Å, and the value predicted using DFT, 11.2 Å. All the peaks shifted to lower 2θ values when the film was heated, suggesting lattice expansion. The temperature dependence of the lattice parameter determined from the XRD measurements is in the Supporting Information (Figure S1), from which a linear expansion coefficient (α = 1/*a* d*a*/d*T*) of 4.7 × 10^–5^ K^–1^ can be determined. This value is typical of halide salts.^24^ No new phases or peaks appeared until 275 °C, and the cubic Cs_2_AgInBr_6_ structure remained stable up to 275 °C. At 275 °C, a new peak at 2θ = 27.1° appeared, which matches the (600) planes of CsAgBr_2_ and (130) planes of InBr_3_. This suggested at least partial decomposition of Cs_2_AgInBr_6_ into Cs_2_AgBr_3_ and InBr_3_ when the film was heated to above 250 °C. This peak remained as the film was heated to even higher temperatures but disappeared at 350 °C. A possible explanation is that the film decomposes and InBr_3_ evaporates at these high temperatures. Cs_2_AgInBr_6_ peak intensities also changed at 350 °C, suggesting that the film became textured with the preferred (400) orientation. When the film was cooled to room temperature, diffraction peaks from Cs_3_In_2_Br_9_, Cs_2_AgBr_3_, and InBr_3_ emerged while Cs_2_AgInBr_6_ peaks disappeared. These changes detected with XRD could also be observed visually as the film’s appearance changed during heating (Supporting Information, Figure S2). The as-deposited film was a semi-transparent light-yellow color and turned into a translucent darker yellow color when heated to 130 °C. This yellow color remained until 250 °C but started fading from 275 °C to 325 °C, and at 350 °C, the film became cloudy. Finally, the film became opaque and white when cooled to room temperature. The changes in the XRD and visual appearance above 250 °C suggested that the Cs_2_AgInBr_6_ film may be destabilized and partially decomposing. It decomposed when cooled to room temperature, as suggested by the XRD and visual appearance. In this experiment, it is unclear if we could not obtain a stable film at room temperature because it was heated above 250 °C.

To determine whether Cs_2_AgInBr_6_ can be stabilized at room temperature, we heated the as-deposited film to 150 °C—a temperature high enough to form cubic Cs_2_AgInBr_6_ but low enough to avoid its decomposition—and cooled it back to room temperature (27 °C). Figure 2a shows the XRD patterns of the film captured every 20 °C as it was heated and cooled. (Data was collected at both 27 °C and 30 °C during temperature ramping up and down. No difference was observed.) As in Figure 1, the as-deposited film comprises ternary compounds and unreacted precursors with no sign of the double perovskite (Figure 2a,b). No change in XRD was observed from 30 °C to 90 °C (Figure 2b). The cubic Cs_2_AgInBr_6_ begins to form at 110 °C, and (220), (222), (400), (422), and (440) peaks emerge in the XRD pattern. Cs_3_In_2_Br_9_ (200) and Cs_2_AgBr_3_ (402) diffractions are still observed, albeit with lower intensities, indicating that the reaction is not yet completed. At 130 °C, almost all ternary phases and precursor diffractions had disappeared except for a small amount of AgBr. There were no significant changes in the diffraction peaks from 130 °C to 150 °C, except for the slight shift to lower 2θ values due to the thermal lattice expansion. The double perovskite structure remained when the film was cooled from 150 °C to room temperature. Thus, Cs_2_AgInBr_6_ double perovskite was successfully obtained at room temperature. We calculate a lattice parameter of 11.00 Å at room temperature consistent with Breternitz et al.^5^ The digital images of the film during and after the heating are shown in the Supporting Information, Figures S3 and S4. The faint yellow color of the as-deposited film remained the same while the film was heated from 30 °C to 90 °C, and there was no change in the XRD patterns. This faint yellow color became more intense and vivid at 110 °C when the double perovskite structure diffractions began appearing in XRD. The film became darker yellow as it was heated to 150 °C, and this color remained when the film was cooled to room temperature.

**Figure 2:**
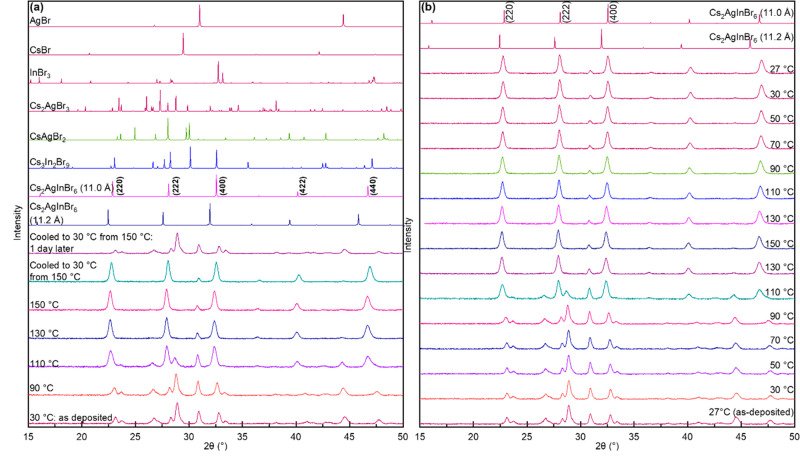
(a) XRD patterns (from bottom to top) of the as-deposited thin film at 30 °C, 90 °C, 110 °C, 130 °C, 150 °C, and cooled to room temperature, along with a film previously heated to 150 °C. Calculated XRD patterns for Cs_2_AgInBr_6_ with *a* = 11.2 Å and *a* = 11.0 Å and potential impurity phases and precursors are also shown for comparison. (b) *In situ* XRD patterns (from bottom to top) of the as-deposited thin film as it is heated to 150 °C and cooled back down to room temperature. Calculated XRD patterns for Cs_2_AgInBr_6_ with *a* = 11.2 Å and *a* = 11.0 Å are shown in (b) for comparison. The film thickness was 510 nm.

However, the Cs_2_AgInBr_6_ double perovskite structure was stable only for 1–2 h, long enough to characterize. It would then gradually decompose into Cs_2_AgBr_3_, Cs_3_In_2_Br_9_, AgBr, and InBr_3_, the same composition as before annealing. This conclusion did not depend on whether the film was annealed in the air or the nitrogen-filled glovebox or whether the film was cooled slowly, stepwise, or naturally (without controlling the temperature or rate of cooling). It also did not matter if the film was kept in the air or the glovebox. For instance, Figure 3 shows the XRD pattern from a film annealed in air at 150 °C for 1 h after that film was kept in the air for 1 day. The XRD pattern is nearly the same as that of the as-deposited film. Figure 3 also shows XRD patterns from films annealed in the glovebox or the air. In all the cases, the Cs_2_AgInBr_6_ double perovskite films eventually decomposed to ternary and binary halides after they were cooled to room temperature. We also checked whether light exposure affected stability because Breternitz et al. reported that the Cs_2_AgInBr_6_ decomposes when exposed to light.^5^Figure 3 also shows the XRD pattern from a Cs_2_AgInBr_6_ film formed by annealing at 150 °C in the air for 1 h after being kept in the dark for a day. The XRD pattern was the same as for the as-deposited film and did not exhibit any of the expected double perovskite peaks. While light exposure may accelerate decomposition, light is certainly not the causing factor. We also checked whether film thickness and stress affected the Cs_2_AgInBr_6_ stability by depositing 100 nm and 1 μm thick films. XRD patterns of these thinner and thicker films recorded *in situ* during the thermal annealing are shown in Figures S5 and S6, respectively. The 100 nm and 1 μm thick Cs_2_AgInBr_6_ films were also unstable and decomposed into Cs_2_AgBr_3_, Cs_3_In_2_Br_9_, AgBr, and InBr_3_ within 1–2 h of cooling to room temperature. Thus, the films appear thermodynamically unstable and eventually decompose independent of the path to room temperature.

**Figure 3:**
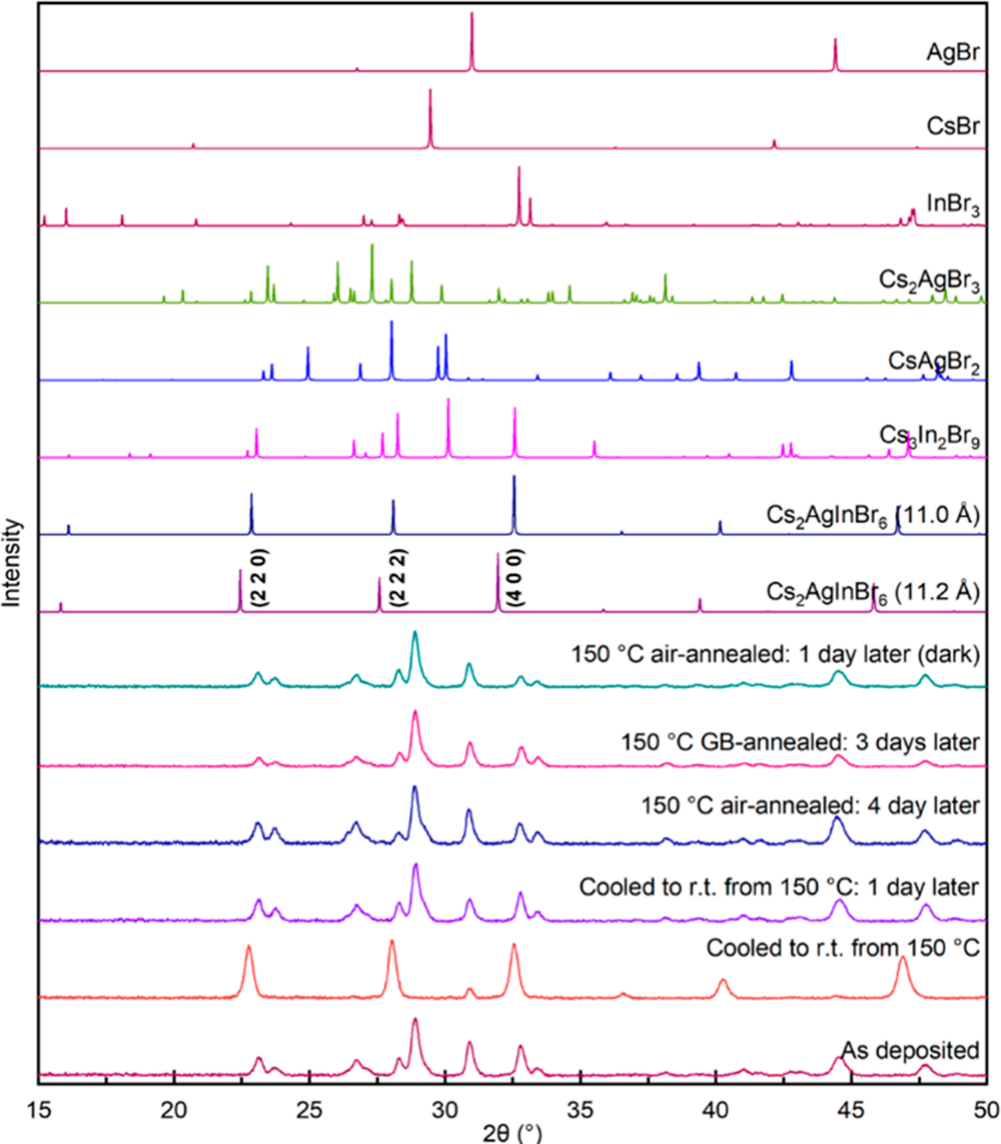
XRD patterns (from the bottom up) from an as-deposited film, a film measured immediately after cooling to room temperature after annealing in air at 150 °C, the same film measured 1 day later and 4 days later, a film annealed in glovebox at 150 °C for 1 h and measured 1 day later, and a film annealed in air at 150 °C for 1 h, kept in the dark, and measured 1 day later. The simulated XRD patterns of Cs_2_AgInBr_6_, Cs_2_AgBr_3_, Cs_3_In_2_Br_9_, CsAgBr_2_, and the precursors are shown for comparison.

No less than nine theoretical studies have examined the stability of Cs_2_AgInBr_6_,^3,6−13^ six of which *predicted it to be stable* by considering its octahedral factor, tolerance factor, formation energy, energy difference with respect to decomposition into ternary and binary bromides, elastic constants, and phonon dispersion (Supporting Information, Table S1). Only three studies raised doubts about the stability of Cs_2_AgInBr_6_.^3,10,11^ Based on free energy calculations, Liang predicted that Cs_2_AgInBr_6_ is unstable with respect to decomposition into Cs_2_AgBr_3_, Cs_3_In_2_Br_9_, and AgBr at 0 K and does not become stable even when the temperature is raised to room temperature, as we observed experimentally.^10^ On the other hand, Menedjhi et al. found from phonon dispersion calculations that Cs_2_AgInBr_6_ is unstable dynamically but stable mechanically.^11^ The simplest argument made by Volonakis et al.,^3^ however, is that the InBr_6_^3–^ octahedra are unstable because the In^3+^ ionic radius is too small (*r*_In^3+^_ = 0.8 Å)^25^ to keep the large Br^–^ cations (*r*_Br^–^_ = 1.96 Å)^25^ away from each other and stabilize their Coulombic repulsion: the octahedral factor, μ = (*r*_In^3+^_/*r*_Br^–^_), is 0.41 at the stability–instability boundary (octahedra are stable for μ > 0.41). Volonakis et al. used the effective Shannon radii in this calculation. This marginal stability is consistent with the Cs_2_AgInBr_6_ crystal structure becoming stabilized as the lattice and octahedra expand with temperatures above room temperature. Applying the octahedral stability factor criteria to a double perovskite is ambiguous because there are two types of octahedra (with Ag and with In). Approaches taken include taking the average Shannon radii of the two cations or using the smallest one. DFT calculations show that Ag–Br and In–Br bond lengths are within 3% of each other. Assuming that they are nearly the same and using the experimentally determined lattice parameter from XRD at room temperature (11.0 Å), we calculate *r*_In^3+^_ + *r*_Ag^+^_ = 1.58 Å and an average octahedral factor of μ = 0.403. To increase this value to μ = 0.41 would require the lattice parameter to increase to 11.05 Å, the value reached at ∼150 °C (see Supporting Information, Figure S1), remarkably close to 130 °C, where we observe the cubic Cs_2_AgInBr_6_ to become stable.

The marginal stability also suggests that Cs_2_AgInBr_6_ may be stabilized at room temperature under tensile strain. Other possibilities for stabilization include substituting some of the Br with Cl. We tried but were unable to stabilize Cs_2_AgInClBr_5_ in preliminary experiments. Clearly, Cs_2_AgInCl_6_ is stable and has been studied,^3^ but at what value of *x* Cs_2_AgIn(Cl_1–*x*_Br_*x*_)_6_ is stabilized remains an open question, and we are addressing this in future work.

As illustrated with digital images in the Supporting Information, Figures S2 and S3, we could tell that the cubic Cs_2_AgInBr_6_ double perovskite had transformed into a mixture of ternary and binary halides from its visual appearance. Immediately after annealing, the film turns clear yellow and remains yellow for approximately 1 h after cooling to room temperature. A day later, the film turns white. Digital photographs of the freshly annealed film and the film a day later are shown in the Supporting Information, Figure S6. Figure 4 quantifies these visual observations and shows the optical absorbance (more correctly extinction) of a 510 ± 10 nm thick Cs_2_AgInBr_6_ film deposited and annealed at 150 °C immediately (within ∼2 min) after cooling to room temperature, 70 min later, and 1 day later. These data are extinction because the measurement includes thin-film interference, reflection, and scattering. The transmission and extinction data exhibit thin-film interference fringes superimposed on absorption that appears to rise at around 800 nm. We corrected the measured extinction by modeling the thin-film interference and refractive index using the method reported by Swanepoel^26^ (see the Supporting Information, Figures S7–S10) and extracted the corrected absorbance of the Cs_2_AgInBr_6_ film, which is also shown in Figure 4. Similar figures for thinner (∼100 nm) and thicker (∼1000 nm) films are shown in the Supporting Information, Figures S11 and S12, respectively. The corrected absorbance of the freshly prepared Cs_2_AgInBr_6_ film started to rise at around 800 nm, and the Tauc plot gives a band gap of 1.57 eV, in agreement with DFT predictions (1.50 eV) by Zhao et al.^8^ and also others (Supporting Information, Table S1). Similar Tauc plots are shown in Figures S9 and S10 for the thinner (100 nm) and thicker films (1055 nm). Averaging the extrapolated band gap values for films with different thicknesses yields a band gap of 1.57 ± 0.1 eV. We tried but could not detect any significant photoluminescence from the film.

**Figure 4:**
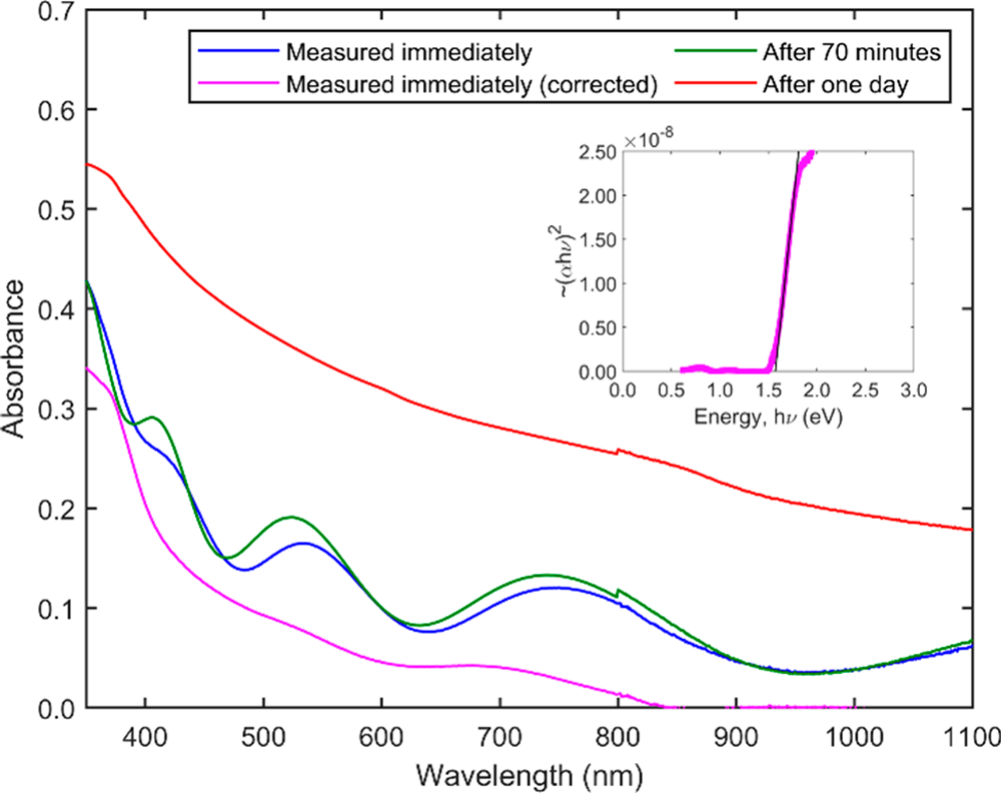
Optical extinction of a 510 ± 10 nm thick Cs_2_AgInBr_6_ film measured immediately (within 2 min) after annealing in the air at 150 °C and cooling to room temperature, 70 min later, and 1 day later. The corrected optical absorption after subtracting interference fringes and surface reflection is also shown. Inset is the direct transition Tauc plot of 510 ± 10 nm thick Cs_2_AgInBr_6_ film measured immediately (within 2 min) after annealing in the air at 150 °C and cooling to room temperature after optical extinction has been corrected for thin film interference. The line is a plausible extrapolation as α → 0 and intersects the energy axis at *x* = 1.57 eV, showing a direct bandgap of 1.57 eV.

The decomposition of the Cs_2_AgInBr_6_ film into ternary and binary phases is accompanied by morphological changes, such as the appearance of larger grains that scatter visible light, which is not surprising. The optical absorbance (extinction) of Cs_2_AgInBr_6_ films began increasing after the first measurement (Supporting Information, Figures S13–S15), indicating that the films start changing after cooling. This decomposition is not easily detected initially with XRD until a significant fraction of the film has decomposed, because XRD is sensitive to the phase volume fraction. Optical measurements are very sensitive to the changes in the film, particularly the morphology and grain sizes. The interference fringes were still discernible initially, but after a day, a strongly scattering background replaced the interference fringes, as shown in Figure 4 for the 510 ± 10 nm thick film. As new phases appear upon decomposition, the grain structure and morphology change. That the phases forming upon decomposition having larger grains than the initial Cs_2_AgInBr_6_ is interesting, but studying the grain sizes of the different phases as Cs_2_AgInBr_6_ decomposes is difficult and beyond the goals and scope of the present study. Scanning electron microscope (SEM) images are consistent with the Cs_2_AgInBr_6_ thin film transforming from a smooth film with 100–200 nm grains to a rough film with grains that are ∼1 μm or larger (Figure 5). With microstructure characterized by a length scale greater than visible light wavelengths, the films become strongly scattering. The elemental composition of the film determined by EDS was 18.9% Cs, 10.5% Ag, 8.8% In, and 61.8% Br, the same as the expected composition of Cs_2_AgInBr_6_ (20% Cs, 10% Ag, 10% In, and 60% Br) within the error of EDS.

**Figure 5:**
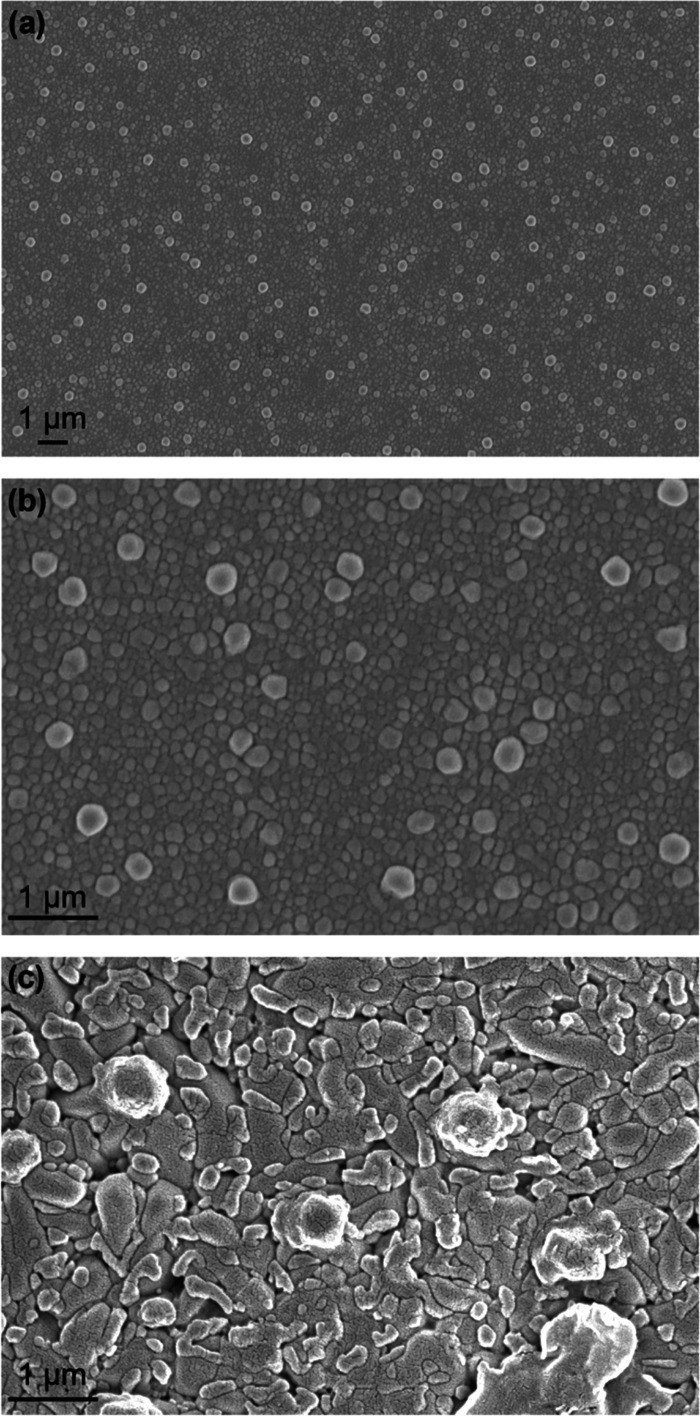
(a, b) SEM images of a ∼500 nm thick Cs_2_AgInBr_6_ film were captured immediately after annealing in the air at 150 °C and cooling to room temperature at two different magnifications. (c) SEM image of a film that has decomposed.

In summary, we formed Cs_2_AgInBr_6_ by evaporating CsBr, AgBr, and InBr_3_ and then annealing between 100 °C and 250 °C. Films were stable in this elevated temperature range but decomposed over 1–2 h to Cs_2_AgBr_3_, Cs_3_In_2_Br_9_, AgBr, and InBr_3_, remaining stable just long enough to be characterized. Furthermore, this decomposition was independent of the film thickness up to 1 μm, illumination, heating, and cooling rates, implicating a thermodynamic instability rooted in the marginal (in)stability of the InBr_6_^3–^ octahedra. The lattice parameter at room temperature is 11.00 ± 0.05 Å and expands upon heating with a linear expansion coefficient of 4.7 × 10^–5^ K^–1^. Optical absorption measurements show a direct band gap of 1.57 ± 0.1 eV. If Cs_2_AgInBr_6_ is to be used for applications, future work should concentrate on approaches for stabilizing it at room temperature.

## Experimental Methods

The Cs_2_AgInBr_6_ thin films were deposited using a six-source physical vapor deposition (PVD) system (Angstrom Engineering) with a base pressure of approximately 10^–8^ Torr. The PVD chamber was enclosed in a nitrogen-filled glovebox. Films were deposited on glass substrates (25 × 25 mm^2^) secured on a rotating stage (10 rpm). Before deposition, the substrates were cleaned by first ultrasonication (Branson 3800) for 30 min in a 50/50 vol% acetone (ACS grade, VWR) and isopropanol (99.5%, VWR) solution, followed by drying in an oven and plasma-cleaning for 30 min in an oxygen plasma cleaner (Harrick Plasma PDC-001-HP). A circulating chiller system (°LAUDA) controlled the substrate stage temperature, and substrates were maintained at 30 °C during deposition. The evaporation rates of each precursor were monitored via individual quartz crystal microbalances (QCMs) with a tooling factor of 39.7 used for CsBr, AgBr, and InBr_3_. Precursors were loaded in alumina ampules and placed in crucible heaters for evaporation. CsBr (99.9%, Acros Organics) and AgBr (99.9%, Beantown Chemical) were baked overnight at 100 °C. InBr_3_ (99%, Sigma-Aldrich) was kept overnight in a vacuum environment at room temperature before use. Precursors CsBr, AgBr, and InBr_3_, were evaporated in the molar stoichiometric ratios (2:1:1) of the target Cs_2_AgInBr_6_ compound (Supporting Information, Table S2). Specifically, the evaporations rates of CsBr, AgBr, and InBr_3_ were 1.28 Å/s, 0.39 Å/s, and 1 Å/s, respectively. The evaporation source temperatures were manipulated to keep the evaporation rates constant and were approximately 570 °C, 600 °C, and 220 °C, respectively. Compared to BiBr_3_ and SbBr_3_, InBr_3_ was much less volatile and evaporated at higher temperatures, making its evaporation rate easier to control.^16,17^ The chamber pressure during the deposition was approximately 10^–6^ Torr. The deposited films were either characterized as deposited or after annealing in the glovebox or the air.

All films were characterized in the air. X-ray diffraction (XRD) patterns were collected using a Bruker AXS D8 DISCOVER GADDS micro-diffractometer equipped with a VÅNTEC-2000 area detector and a Cu source (λ = 1.54178 Å) operated at 40 kV and 40 mA. The scans were area averaged by oscillating the sample laterally with scan oscillation amplitudes across an area of 2 × 2 mm^2^. The diffraction patterns at high temperatures were recorded using a heated sample holder in the air under ambient conditions. High-resolution images of film topography were recorded in a MERLIN Carl Zeiss field emission scanning electron microscope at 5 kV acceleration voltage and 110 pA beam current under an in-lens (annular secondary electron) detector and an SE2 (Everhart-Thornley type) detector. Film elemental compositions were determined using energy-dispersive X-ray spectroscopy (EDS) at 20 kV acceleration voltage and 2000 pA beam current. The EDS measurements collected over 500 nm × 500 nm areas at different locations were averaged and reported. Film optical absorption between 300 and 2000 nm was measured in a UV-vis-NIR spectrophotometer (Agilent Technologies Cary 5000) with a blank glass substrate as the reference. The film thicknesses were determined by fitting the interference fringes in the optical transmission. The values were consistent with cross-sectional SEM measurements (Supporting Information, Figures S8 and S16).

## Supporting Information Available

Additional tables and figures showing temperature dependence of the lattice parameter, digital images of the films as a function of temperature, XRD of films with different thicknesses, optical extinction (absorption) and the details of interference fringe correction methods, optical extinction as a function of time, summary table of prior studies reporting stability, lattice parameter, and band gap, and further experimental details (PDF)
