# Supplementary material for: Stability of the Halide Double Perovskite Cs2AgInBr6
Source: J Phys Chem Lett. 2023 Mar 21;14(12):3000–6. doi: 10.1021/acs.jpclett.3c00303 (PMC10068733; doi:10.1021/acs.jpclett.3c00303)
Supplement: Supplementary file 1 — jz3c00303_si_001.pdf [file jz3c00303_si_001.pdf]

Supporting information

Stability of the Halide Double Perovskite $Cs_2AgInBr_6$

*Yukun Liu, Iver J. Cleveland, Minh N. Tran, and Eray S. Aydil**

Department of Chemical and Biomolecular Engineering, New York University, Tandon School of Engineering, Brooklyn, New York 11201, USA

Corresponding Author

*E-mail: aydil@nyu.edu

A. Temperature dependence of the $Cs_2AgInBr_6$ lattice parameter

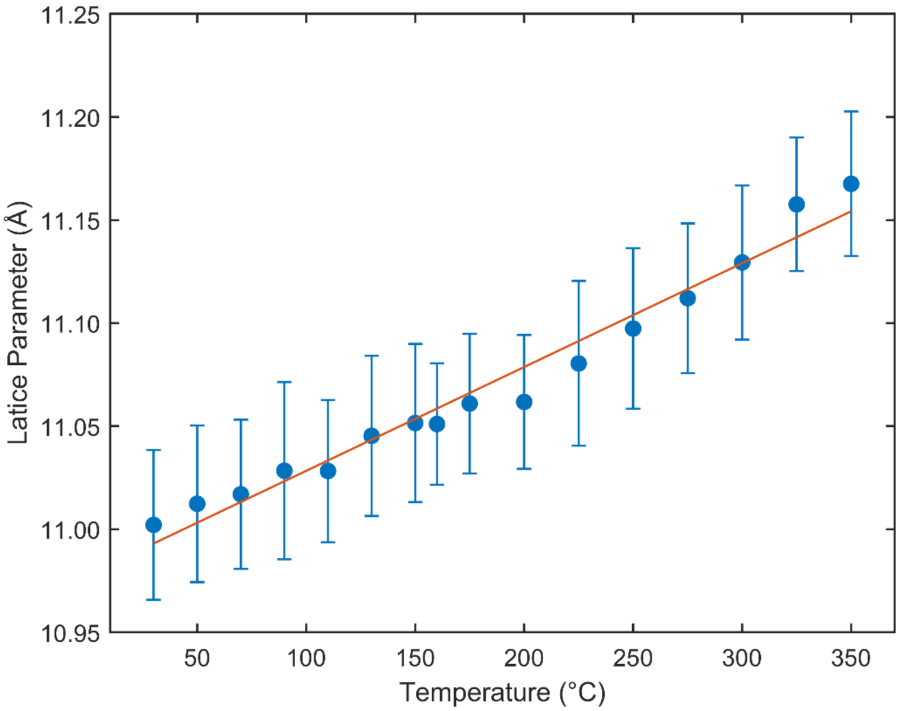

Figure S1. $Cs_2AgInBr_6$ lattice parameters determined from XRD measurements at room temperature, 50 °C, 70 °C, 90 °C, 110 °C, 130 °C, 140 °C, 150 °C, 160 °C, 175 °C, 200 °C, 225 °C, 250 °C, 275 °C, 300 °C, 325 °C, and 350 °C. Standard deviations are shown as error bars. The line is the linear least squares fit of the data ($a = 5\times10^{-4}T + 10.84$) where T is in K. The linear expansion coefficient ($\alpha = 1/a\ da/dT$) calculated from $m = da/dT$ over this range is $4.52\times10^{-5}K^{-1}$.

B. Thin film appearance as a function of temperature

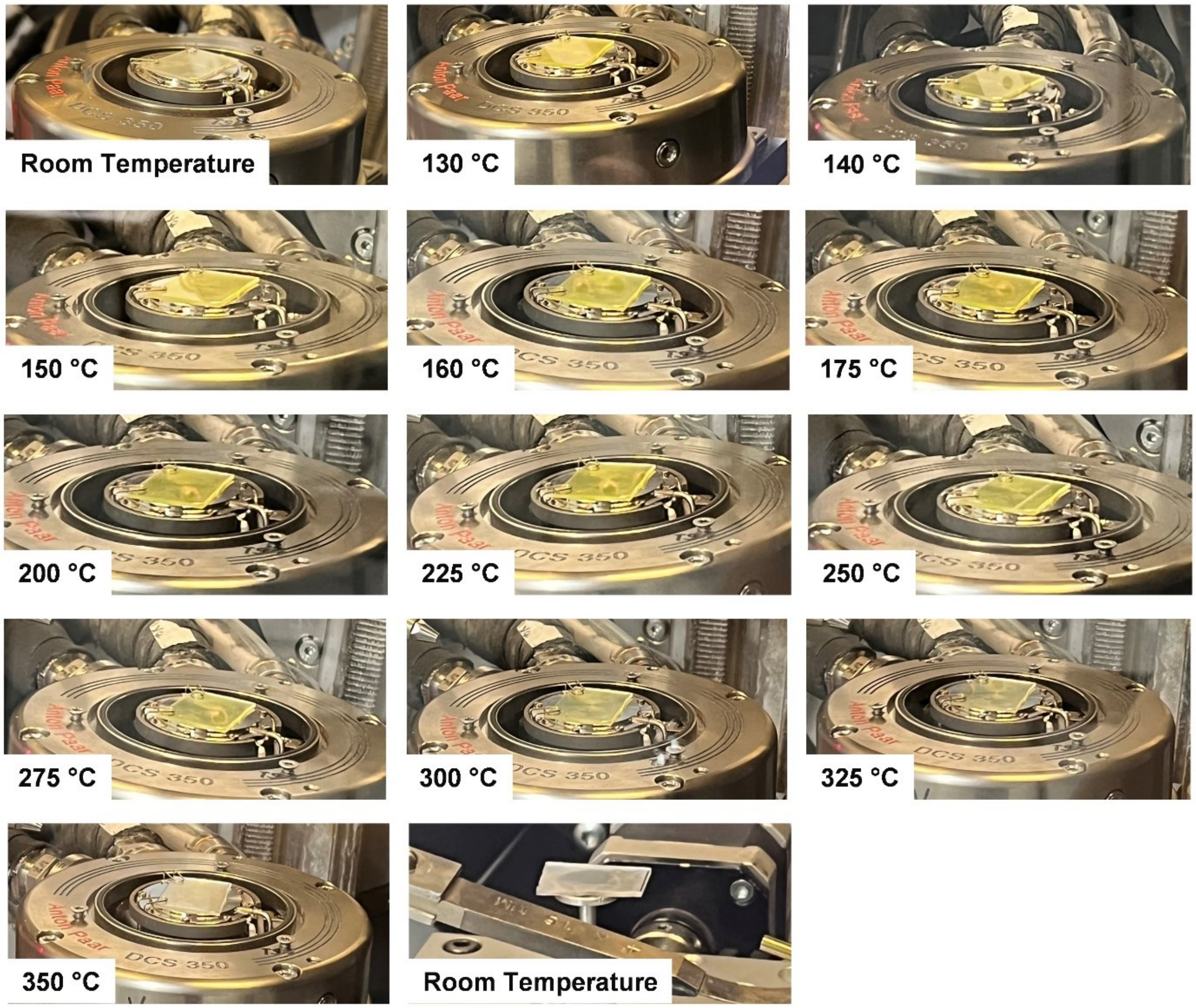

Figure S2. Digital images of the film on glass from room temperature to 350 °C and back down to room temperature at the end of heating to 350 ºC.

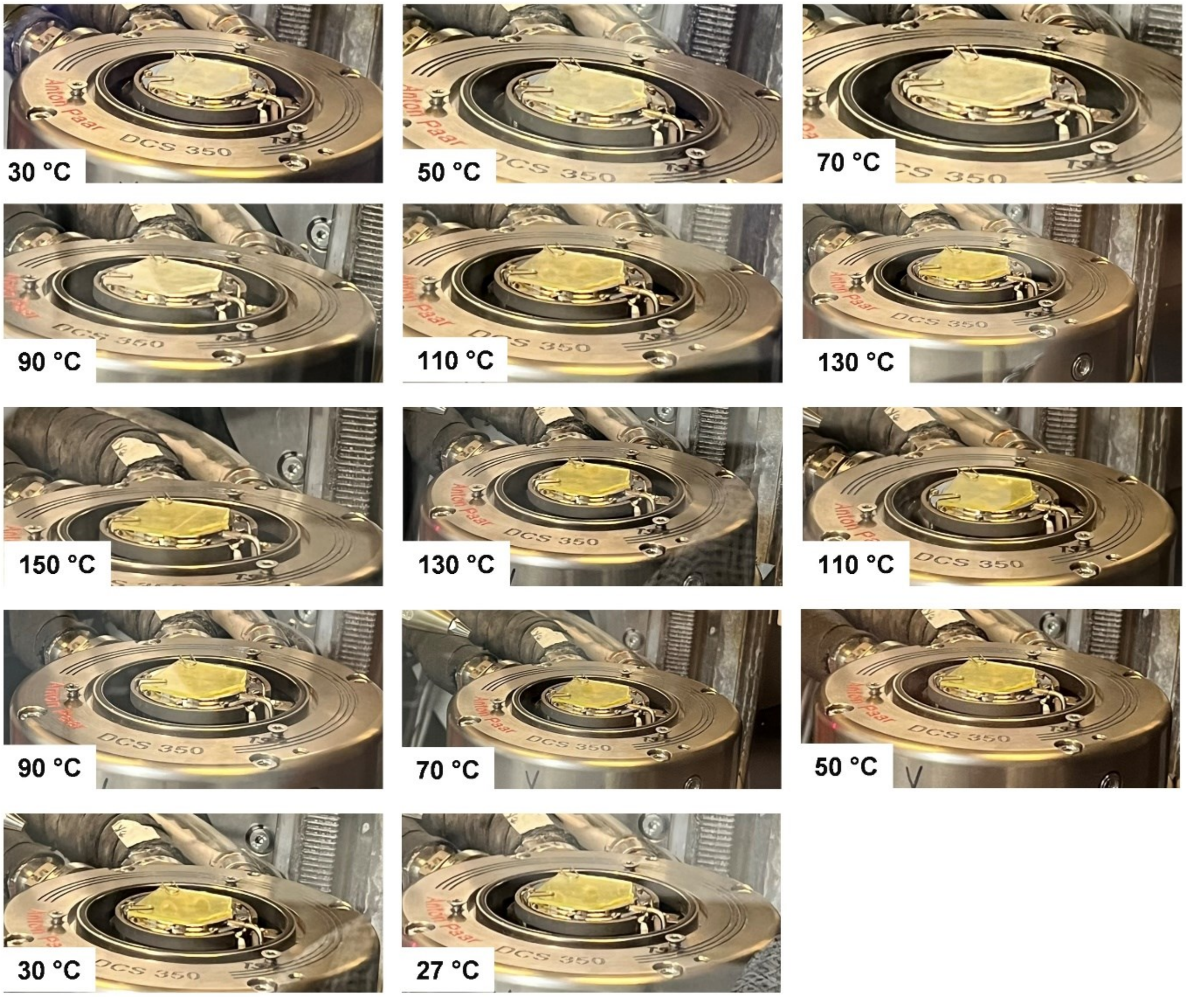

Figure S3. Digital images of the film on glass at 30 °C, 50 °C, 70 °C, 90 °C, 110 °C, 130 °C, 150 °C, 130 °C, 110 °C, 90 °C, 70 °C, 50 °C, and 30 °C during the XRD measurements.

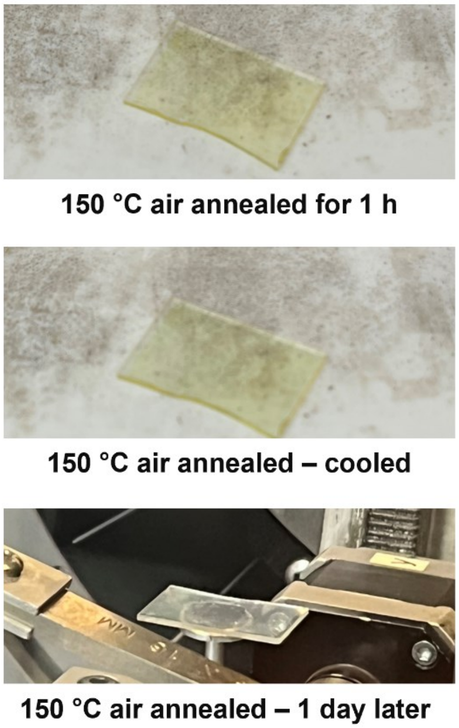

Figure S4. Digital images of the freshly annealed film (150 °C in the air for 1 hour) and the same film after a day.

C. X-ray Diffraction Patterns of Films with Different Thicknesses

Films thinner and thicker than 500 nm (~100 nm and ~1000 nm) were deposited by decreasing and increasing the deposition time under identical deposition conditions to study whether film stress and thickness affect the material stability. The deposition times were 5 minutes, 30 minutes, and 55 minutes for the ~100 nm, ~ 500 nm, and ~ 1000 nm thick films, respectively. Figure S5a shows the XRD patterns from a ~1000 nm thick film as deposited at room temperature, after heating to 90 ºC, 110 ºC, and 150 ºC, immediately after cooling to room temperature after this heating, after a few hours the same day and after one day. Figure S5b shows the XRD patterns during in situ annealing as a function of temperature. As with the 500 nm film, the as-deposited ~1000 nm thick film comprises Cs_2AgBr_3, $Cs_3In_2Br_9$, AgBr, and $InBr_3$, indicating only partial reaction between CsBr and AgBr and $InBr_3$ during the deposition to form the ternary phases with no evidence of $Cs_2AgInBr_6$ formation. $Cs_2AgInBr_6$ forms upon heating to 150 ºC, which is stable upon cooling to room temperature but only for a few hours (typically less than 4 hours). Eventually, the film decomposes to Cs_2AgBr_3, $Cs_3In_2Br_9$, and $InBr_3$.

Figure S6a and S6b show the same for the thinner (~100 nm) film. The detector and the incident X-ray were positioned at lower incident angles (θ_1 =5°, θ_2 =27°) to increase the sampling volume and, thus, the peak intensities, which is responsible for the peak broadening in Figure S6. The objective was to ascertain the film's stability. As with the thicker films, $Cs_2AgInBr_6$ double perovskite started to form at 110 °C, and the reaction was completed at 150 °C. When the films are measured immediately after cooling to room temperature, $Cs_2AgInBr_6$ is still observed. However, the structure is only stable for less than 4 hours and decomposes completely after a day. In conclusion, film thickness and stress do not seem to impact material stability in the film thickness range of 100 to 1000 nm.

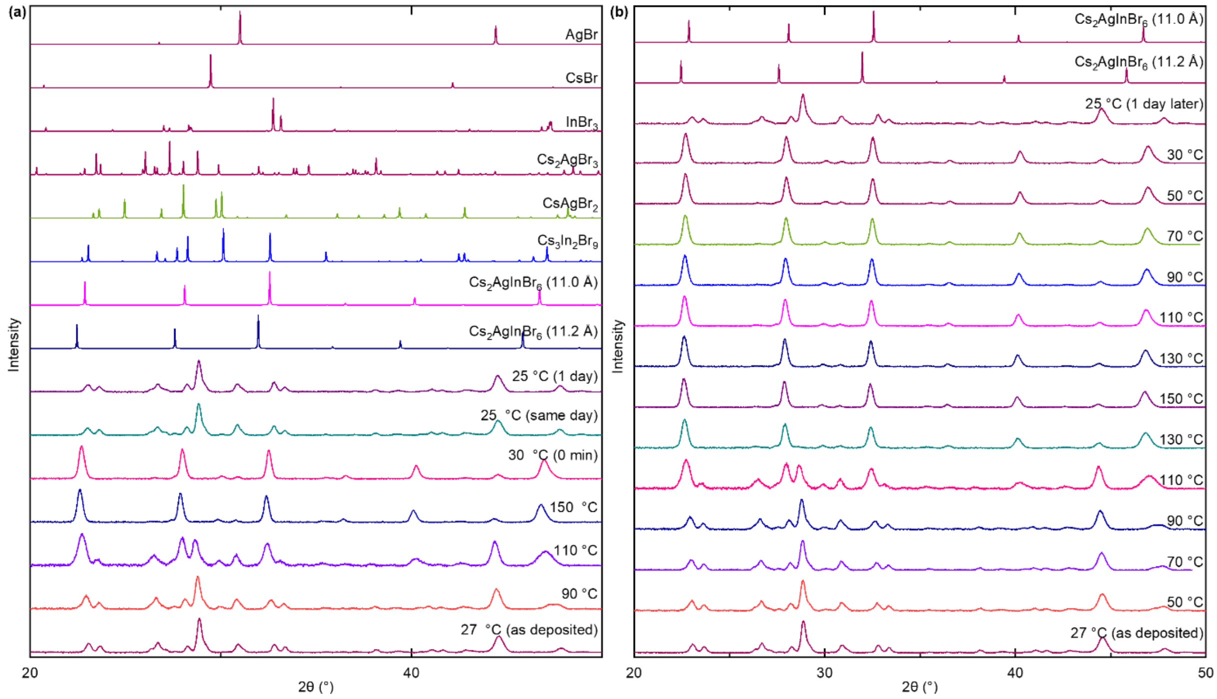

Figure S5. (a) *In situ* XRD measurements of the ~1000 nm thick film (from bottom to top) as deposited at room temperature, at 90 °C, at 110 °C, at 150 °C, measured immediately when cooled to 30 °C (denoted as 0 min), after storing at room temperature for a few hours, and after storing at room temperature for a day. Simulated XRD patterns of $Cs_2AgInBr_6$ (using a = 11.2 Å and a = 11.0 Å) and precursor are also shown as references. (b) *In situ* XRD measurements of the ~1000 nm thick film as it is heated stepwise from room temperature to 150 °C and then cooled stepwise from 150 °C to room temperature, and after storing at room temperature for a day, along with simulated XRD patterns of $Cs_2AgInBr_6$ (using a = 11.2 Å and a = 11.0 Å). The film thickness determined by fitting the thin film interference fringes in the optical transmission was 1055 nm.

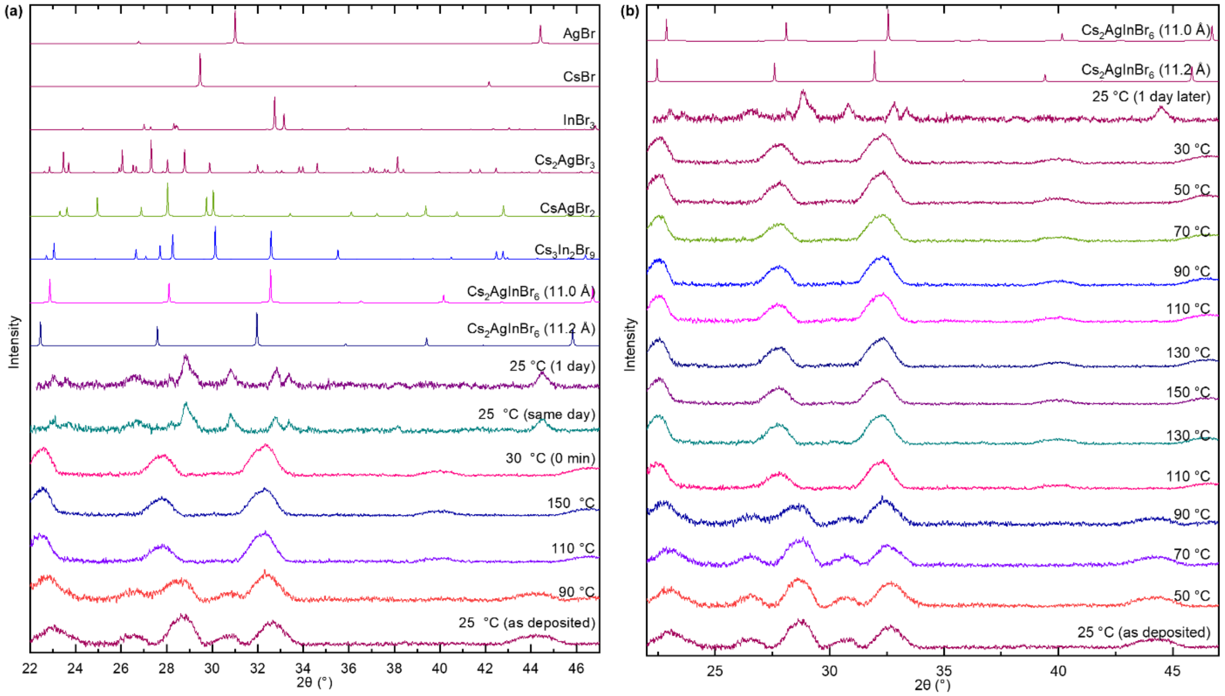

Figure S6. (a) *In situ* XRD measurements of the ~100 nm thick film (from bottom to top) as deposited at room temperature, at 90 °C, at 110 °C, at 150 °C, measured immediately when cooled to 30 °C (denoted as 0 min), after storing at room temperature for a few hours, and after storing at room temperature for a day. Simulated XRD patterns of $Cs_2AgInBr_6$ (using a = 11.2 Å and a = 11.0 Å) and precursor are also shown as references. (b) *In situ* XRD measurements of the ~100 nm thick film as it is heated stepwise from room temperature to 150 °C and then cooled stepwise from 150 °C to room temperature, and after storing at room temperature for a day, along with simulated XRD patterns of $Cs_2AgInBr_6$ (using a = 11.2 Å and a = 11.0 Å).

D. $Cs_2AgInBr_6$ Films' Optical Properties

The interference fringes are modeled in the transmission spectrum using a method reported by Swanepoel.[1] We calculated the thin film interference fringes using an optical model of an absorbing thin film on a thick finite substrate (Figure S7). We then subtracted the calculated fringes from the measured extinction to determine the corrected film absorbance. First, the baseline of the measured transmission is set to

$$T_s = \frac{2s}{s^2+1} \tag{S10}$$

by subtracting, from the measured extinction spectrum, the difference between the measured value of the transmission at the peak of the fringe that appears at the largest λ and T_s. In this equation, s is the glass refractive index (1.5). The transmission is calculated from

$$T = \frac{A'x}{B' - C'x\cos\varphi + D'x^2} \tag{S11}$$

where

$$A' = 16\, s\, (n^2 + k^2) \tag{S12}$$

$$B' = [(n+1)^2 + k^2][(n+1)(n+s^2) + k^2] \tag{S13}$$

$$C' = [(n^2 - 1 + k^2)(n^2 - s^2 + k^2) - 2k^2(s^2+1)]\, 2\cos\varphi - k[2(n^2 - s^2 + k^2) + (s^2+1)(n^2 - 1 + k^2)]\, 2\sin\varphi \tag{S14}$$

$$D' = [(n-1)^2 + k^2][(n-1)(n-s^2) + k^2] \tag{S15}$$

$$\varphi = \frac{4\pi n d}{\lambda} \tag{S16}$$

$$x = \exp(-\alpha d) \tag{S17}$$

$$\alpha = \frac{4\pi k}{\lambda} \tag{S18}$$

and where n and k are the real and imaginary components of the film's complex refractive index ($\tilde{n} = n - ik$), and d is the film thickness. n is calculated as follows. First, fringe maxima, T_M, and minima, T_m, are identified, and their λ dependence is fit to a polynomial. Features suspected to be absorption peaks in the absorbing region are avoided, and only the minima and maxima above the nonabsorbing region (in this case > 800 nm) are used. n in the nonabsorbing region is calculated from

$$n = \sqrt{M + \sqrt{M^2 - s^2}} \tag{S19}$$

where

$$M = \frac{2s}{T_m} - \frac{s^2+1}{2} \tag{S20}$$

and n in the absorbing region is calculated from

$$n = \sqrt{N + \sqrt{N^2 - s^2}} \tag{S21}$$

where

$$N = 2s\,\frac{T_M - T_m}{T_M T_m} - \frac{s^2+1}{2}. \tag{S22}$$

The corrected absorbance is calculated using

$$A = -\log(T_{exp} - T), \tag{S23}$$

where T_{exp} is the experimentally measured baseline corrected transmission.

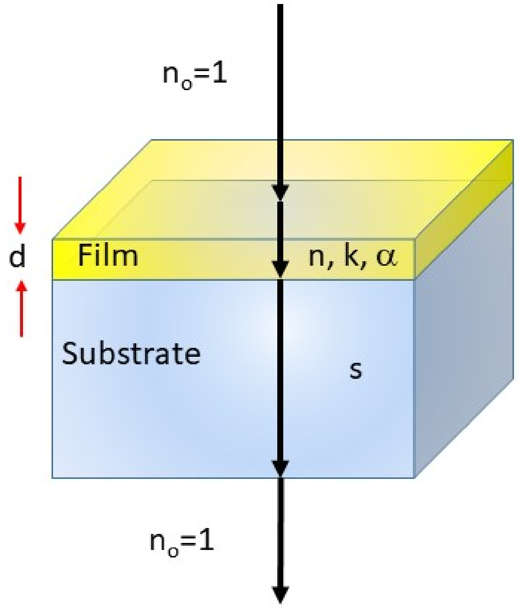

Fig. S7 Schematic of the optical model used to calculate the thin film interference fringes and correct the measured transmission (extinction).

Figure S8 shows the measured and corrected absorbance (transmission) and fitted refractive index for an example film. This film was deposited as described in the main text Experimental Methods Section with a target thickness of 500 nm. It was annealed in air post-deposition by heating it slowly (~10 min) to 150 °C and cooling it slowly (~40 min) to room temperature. The absorbance (transmission) was measured immediately (within 2 minutes) after cooling to room temperature. Figure S8a shows the measured and modeled transmission (using Swanepoel's method). The modeled fringes were subtracted from the experimental transmission

spectrum, and the remaining was converted to absorbance. Figure S8b shows the absorbance spectrum before (as-measured) and after correction. The corrected film absorption starts to increase at 800 nm (1.55 eV). The film thickness and refractive index that best fit the fringes were 510 nm and 1.87 ±0.03, respectively (Figure S8c). The values of the refractive index below 800 nm are extrapolations. The refractive index is expected to rise slightly as absorption begins. Such small rises have only a small effect on the thickness but may affect the locations of the interference fringes, leading to imperfect subtraction. For this reason, the features in the absorption spectra should be interpreted with caution as they may result from small residual fringes remaining after subtraction.

Figure S9 shows the optical absorbance of a 100 nm thick $Cs_2AgInBr_6$ film immediately (within 2 minutes) after cooling to room temperature, 70 minutes later, and one day later. Figure S10 shows the same for a 1055 nm thick film. The corrected absorbance of the 1055 nm thick $Cs_2AgInBr_6$ film is also shown in Figure S10. The 100 nm thick film data was not corrected because there were no complete fringes. Like the 510 nm thick film, the transmission and extinction of the 1055 nm thick film exhibit thin film interference fringes superimposed on absorption that appears to rise at around 800 nm.

The insets in S9 and S10 show Tauc plots. Only the data near where absorption goes to zero (A or $\alpha \rightarrow 0$). Sometimes, different lines could be drawn to the data. See, for example, Fig S9. These different extrapolations gave slightly different values, and this variation is the reason for reporting the average of these values with ±0.1 eV error bars.

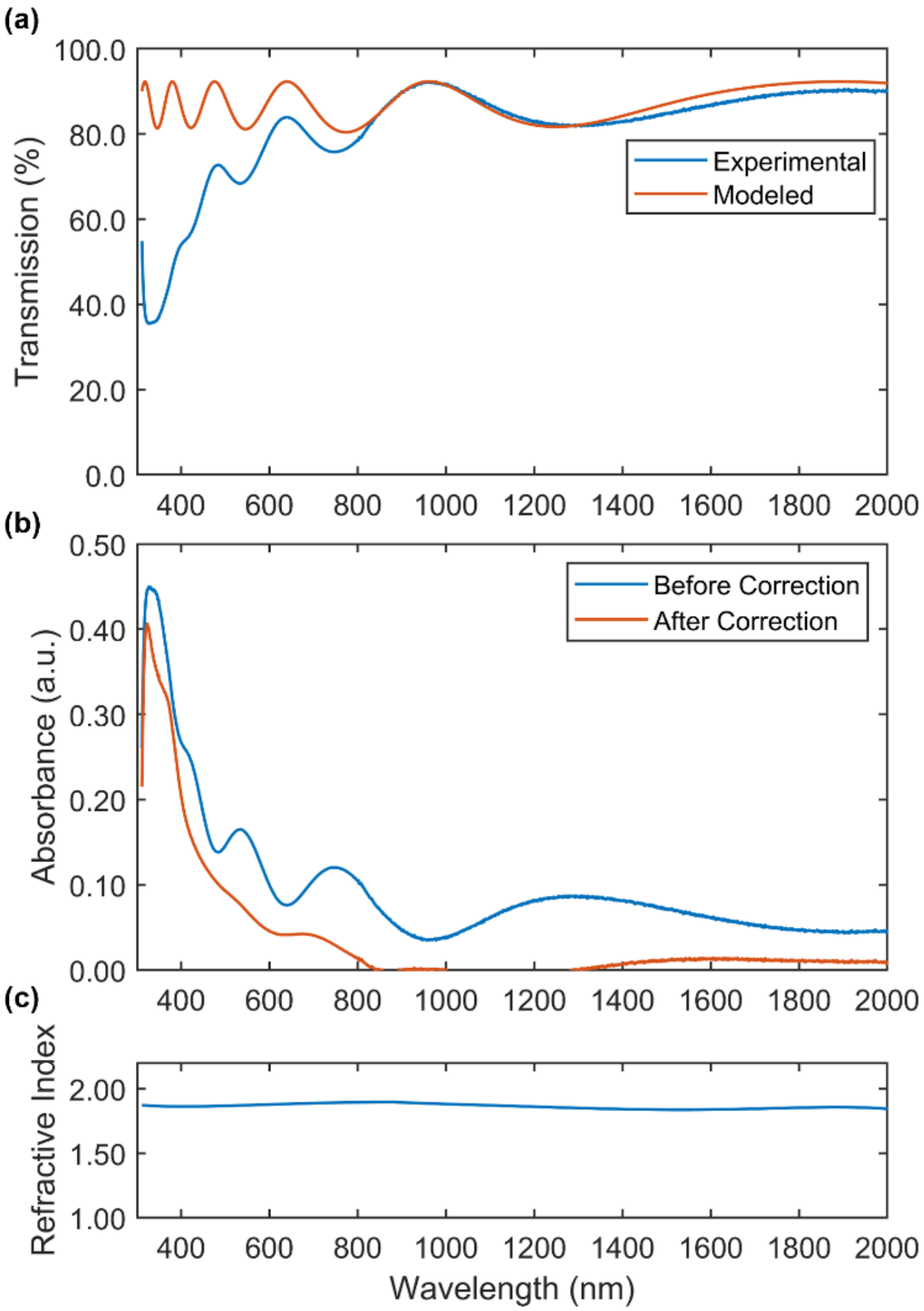

Fig. S8. (a) The experimentally measured and modeled transmission spectra of a 510 nm thick $Cs_2AgInBr_6$ film. (b) Absorbance spectra of the same film before and after subtracting the modeled interference fringes. Panel (c) shows the refractive index used to fit the interference fringes.

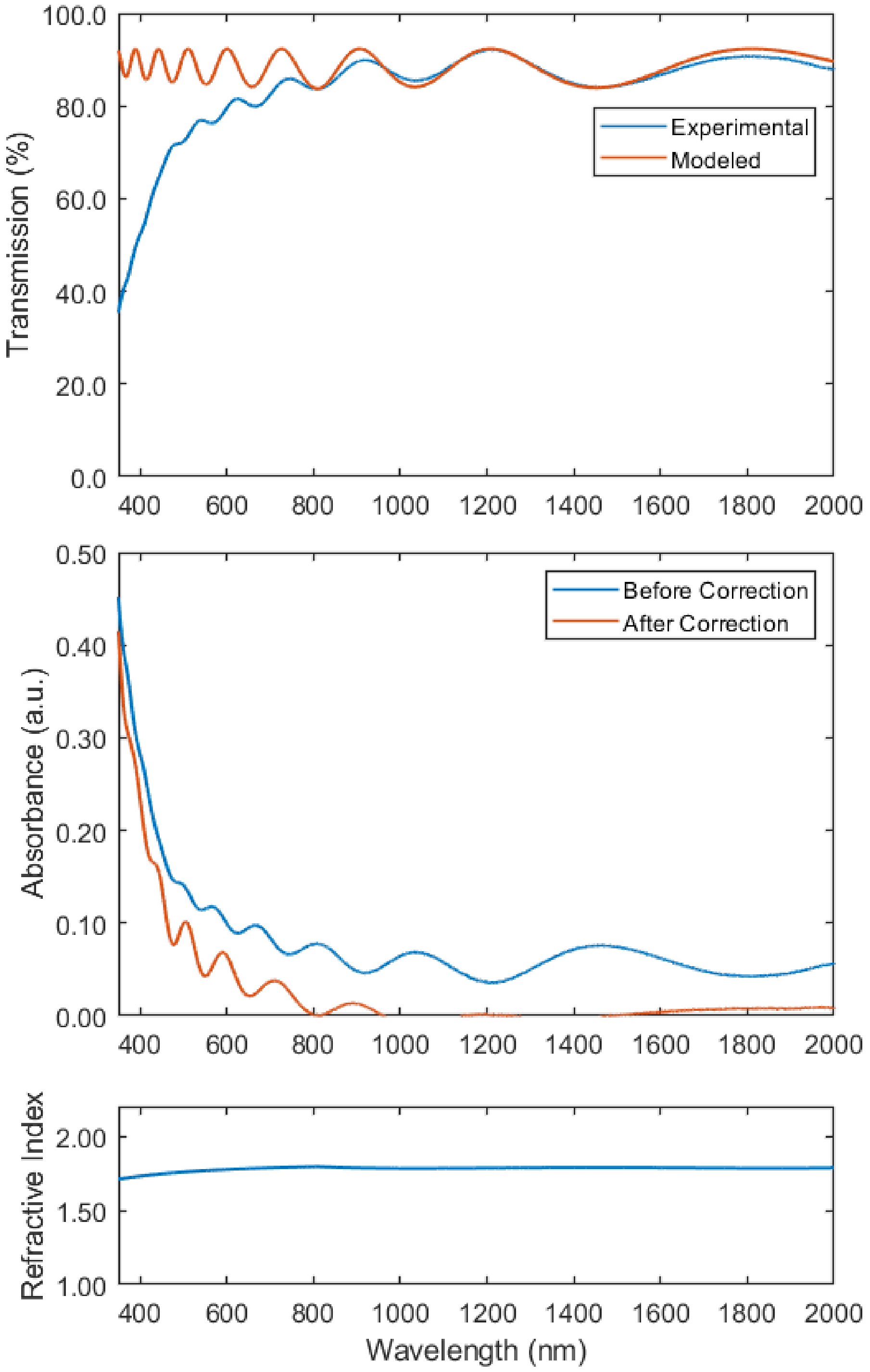

Fig. S9. (a) The experimentally measured and modeled transmission spectra of a ≈ 1 μm thick $Cs_2AgInBr_6$ film. (b) Absorbance spectra of the same film before and after subtracting the modeled interference fringes. Panel (c) shows the refractive index used to fit the interference fringes.

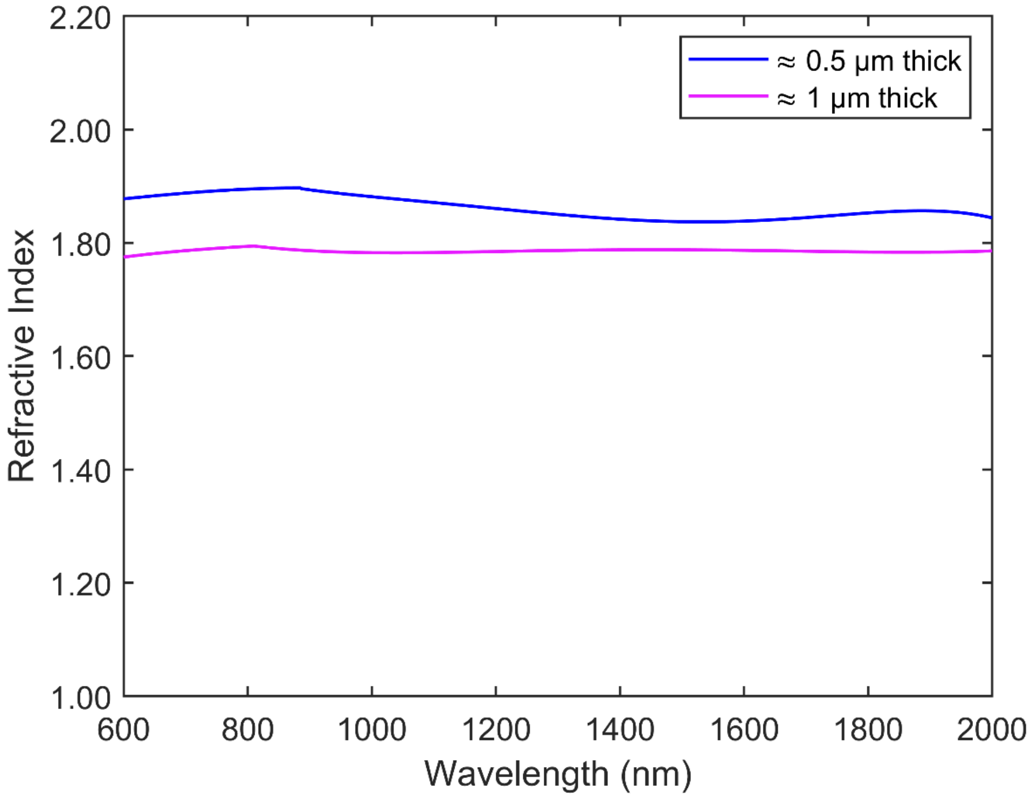

Fig. S10. The refractive index fits to the thin film interference fringes in the ≈ 0.5 μm and ≈ 1 μm thick films.

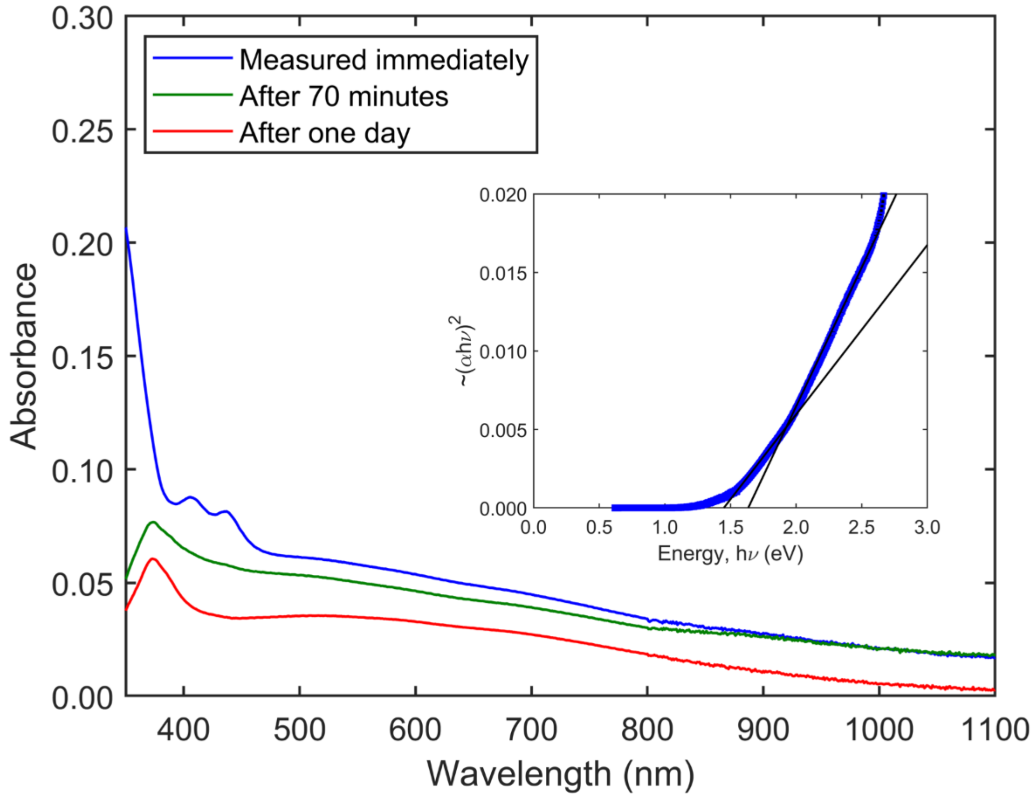

Fig. S11. Optical absorbance (more correctly extinction) of a 100 nm thick $Cs_2AgInBr_6$ film deposited and annealed at 150 ºC immediately (within 2 minutes) after cooling to room temperature, 70 minutes later, and one day later. Inset is the direct transition Tauc Plot of the 100 nm thick $Cs_2AgInBr_6$ film deposited and annealed at 150 ºC measured immediately (within 2 minutes) after cooling to room temperature. The two lines are different choices of extrapolations and intercept the energy axis at 1.45 eV or 1.64 eV, showing direct bandgaps between 1.46 to 1.64 eV.

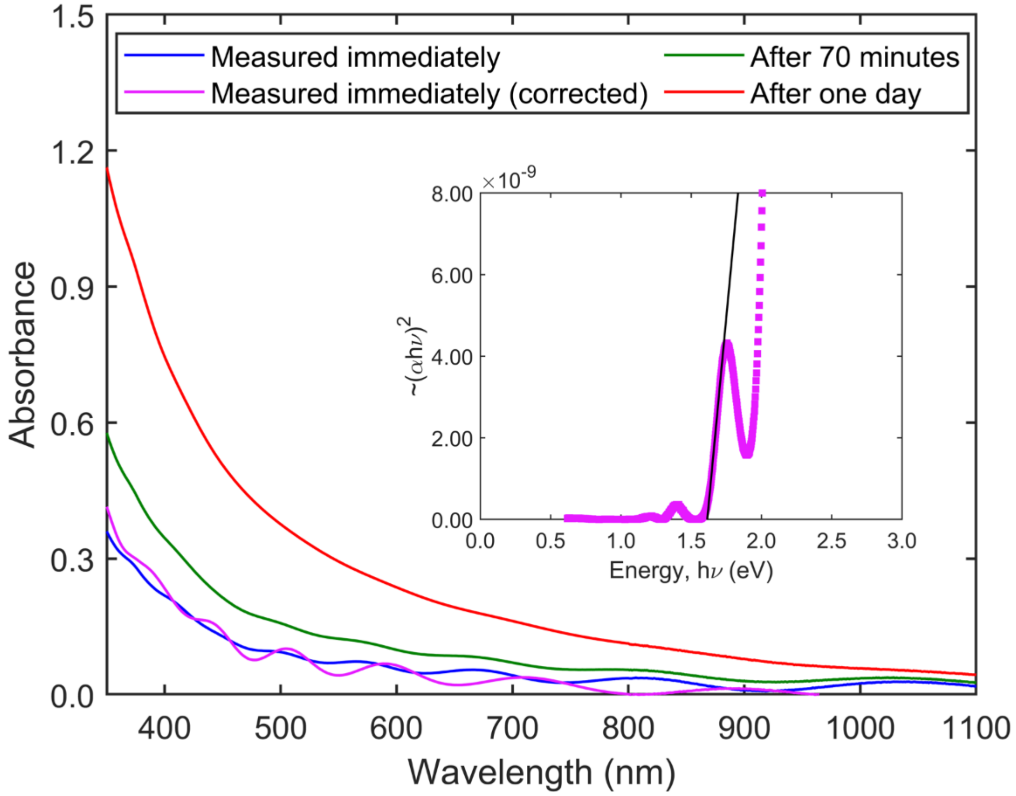

Fig. S12. Optical absorbance (more correctly extinction) of a 1055 nm thick $Cs_2AgInBr_6$ film deposited and annealed at 150 ºC immediately (within 2 minutes) after cooling to room temperature, 70 minutes later, and one day later. The corrected absorbance is also shown. Inset is the direct transition Tauc Plot of the 1055 nm thick $Cs_2AgInBr_6$ film deposited and annealed at 150 ºC measured immediately (within 2 minutes) after cooling to room temperature. The line in the offset is a plausible extrapolation and intercepts the energy axis at 1.60 eV. The small peaks below 1.5 eV in the inset are residual due to incomplete subtraction of the interference fringes.

Table S1. A summary of prior work on determining the stability, band gap, and lattice parameter of $Cs_2AgInBr_6$. Abbreviations for functionals: B3LYP: Becke 3-Parameter, Lee, Yang and Parr; HSE06: Heyd-Scuseria-Ernzerhof; LDA-PAW: Local Density Approximation Projected Wave Augmented Wave; PBE0: Perdew–Burke-Ernzerhof; SOC: Spin-Orbit Coupling; VPSIC: Variational pseudo-self interaction correction

Bandgap (eV)	Direct or Indirect	Lattice Parameter (Å)	Stability	Method	Reference
1.25	Direct	N/A	Stable	HSE06+SOC	Dai *et al.*[2]
1.33	Direct	11.20	Stable	HSE06+SOC	Xu *et al.*[3]
1.50	Direct	11.156	Stable	HSE06+SOC	Zhao *et al.*[4]
0.58	Direct	11.43	Stable	PAW-LDA	Zhang *et al.*[5]
1.17	Direct	10.93	Stable	VPSIC	Liu *et al.*[6]
1.64	Direct	N/A	Unstable	HSE06	Liang *et al.*[7]
1.427	Direct	11.23	Mechanically Stable Thermodynamically Unstable	B3LYP	Menedjhi *et al.*[8]
1.47	Direct	11.19	Stable	HSE06	Wang *et al.*[9]
1.47	Direct	11.20	Stable	HSE06	Li *et al.*[10]
1.7	Direct	10.74	Maybe	HSE-PBE0	Volonakis *et al.*[11]
2.36	Direct	10.997(5)	Unstable under illumination	Experimental	Breternitz *et al.*[12]
1.57	Direct	11.00±0.05	Thermodynamically Unstable	Experimental	This Work

E. $Cs_2AgInBr_6$ optical extinction as a function of time

Optical absorbances of $Cs_2AgInBr_6$ films were measured as a function of time as indicators of $Cs_2AgInBr_6$ stability at room temperature. Figures S11-S13 show the optical extinction of the 1050 nm, 510 nm, and 100 nm thick $Cs_2AgInBr_6$ films as a function of time at room temperature after they were annealed in the air at 150 °C and then cooled to room temperature in the air.

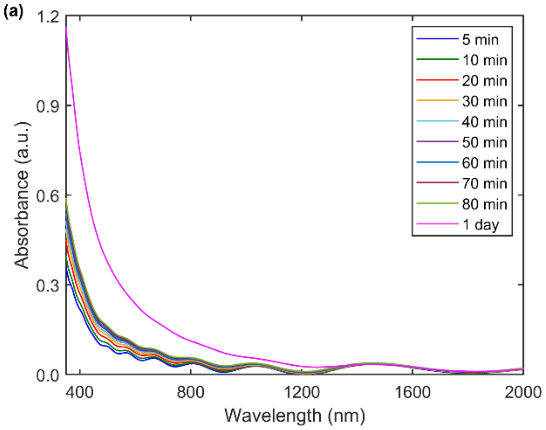

Figure S13. The optical absorbance of the thicker $Cs_2AgInBr_6$ film (1050 nm) stored at room temperature as a function of time after it has been annealed in air at 150 ºC and cooled to room temperature in the wavelength ranges of (a) 300 to 2000 nm and (b) 300 to 800 nm expanded for clarity.

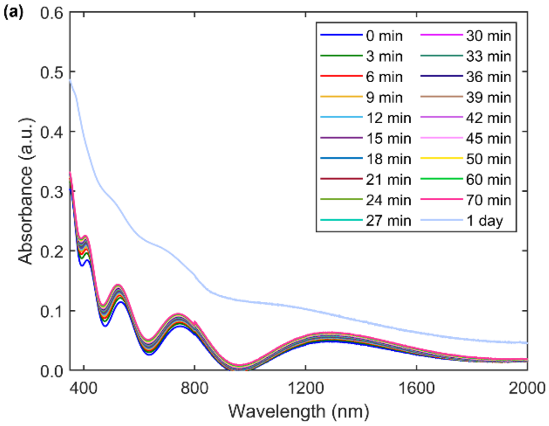

Figure S14. The optical absorbance of the 510±10 nm thick $Cs_2AgInBr_6$ film stored at room temperature as a function of time after it has been annealed in air at 150 ºC and cooled to room temperature in the wavelength ranges of (a) 300 to 2000 nm and (b) 300 to 800 nm expanded for clarity.

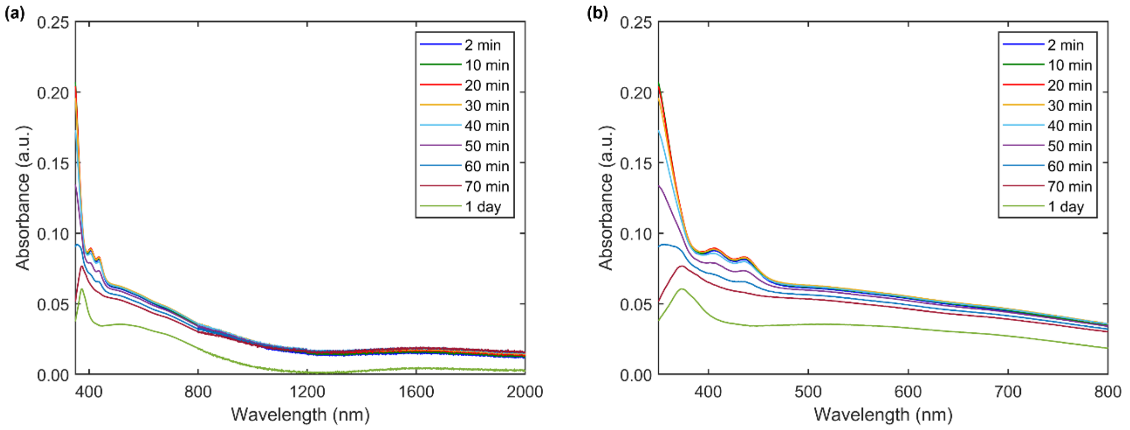

Figure S15. The optical absorbance of the thinner $Cs_2AgInBr_6$ film (~100 nm) stored at room temperature as a function of time after it has been annealed in air at 150 ºC and cooled to room temperature in the wavelength ranges of (a) 300 to 2000 nm and (b) 300 to 800 nm expanded for clarity.

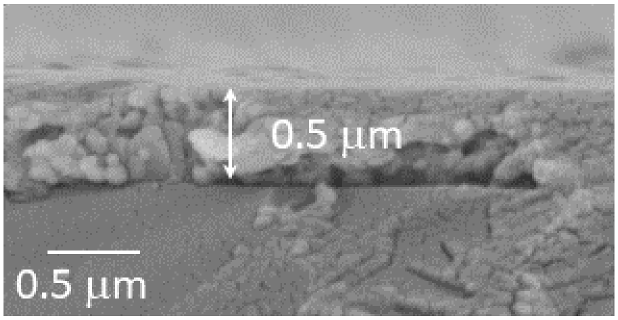

Figure S16. SEM of a $Cs_2AgInBr_6$ film determined to be 510 nm by fitting the thin film interference fringes in the optical transmission.

F. Experimental Details

Table S2. Properties used in the calculation of the CsBr, AgBr, and $InBr_3$ deposition rates (at the substrate plane).

Compound	Molecular Weight (g/mole)	Density (g/cm^3)	Deposition Rate (Å/s)	Flux (mol/cm^2 s)	Flux Ratios
CsBr	212.81	4.44	1.28	2.67×10^{-10}	2
AgBr	187.77	6.473	0.39	1.34×10^{-10}	1
$InBr_3$	354.53	4.74	1.00	1.34×10^{-10}	1

References

(1) Swanepoel, R. Determination of the Thickness and Optical Constants of Amorphous Silicon. *J. Phys. E.* **1983**, *16*, 1214–1222.

(2) Dai, J.; Ma, L.; Ju, M.; Huang, J.; Zeng, X. C. In- and Ga-Based Inorganic Double Perovskites with Direct Bandgaps for Photovoltaic Applications. *Phys. Chem. Chem. Phys.* **2017**, *19*, 21691–21695.

(3) Xu, J.; Liu, J.-B.; Liu, B.-X.; Huang, B. Intrinsic Defect Physics in Indium-Based Lead-Free Halide Double Perovskites. *J. Phys. Chem. Lett.* **2017**, *8*, 4391–4396.

(4) Zhao, X.-G.; Yang, D.; Sun, Y.; Li, T.; Zhang, L.; Yu, L.; Zunger, A. Cu–In Halide Perovskite Solar Absorbers. *J. Am. Chem. Soc.* **2017**, *139*, 6718–6725.

(5) Zhang, Z.; Su, J.; Hou, J.; Lin, Z.; Hu, Z.; Chang, J.; Zhang, J.; Hao, Y. Potential Applications of Halide Double Perovskite Cs_2AgInX_6 (X = Cl, Br) in Flexible Optoelectronics: Unusual Effects of Uniaxial Strains. *J. Phys. Chem. Lett.* **2019**, *10*, 1120–1125.

(6) Liu, F.; Marongiu, D.; Pau, R.; Sarritzu, V.; Wang, Q.; Lai, S.; Lehmann, A. G.; Quochi, F.; Saba, M.; Mura, A.; Bongiovanni, G.; Mattoni, A.; Caddeo, C.; Bosin, A.; Filippetti, A. Ag/In Lead-free Double Perovskites. *EcoMat* **2020**, *2*, e12017.

(7) Liang, Y. Exploring Inorganic and Nontoxic Double Perovskites $Cs_2AgInBr6_{(1-x)}Cl_{6x}$ from Material Selection to Device Design in Material Genome Approach. *J. Alloys Compd.* **2021**, *862*, 158575.

(8) Menedjhi, A.; Bouarissa, N.; Saib, S.; Bouamama, K. Halide Double Perovskite $Cs_2AgInBr_6$ for Photovoltaic's Applications: Optical Properties and Stability. *Optik (Stuttg).* **2021**, *243*, 167198.

(9) Wang, K.; He, Y.; Zhang, M.; Shi, J.; Cai, W. Promising Lead-Free Double-Perovskite Photovoltaic Materials $Cs_2MM'Br_6$ (M = Cu, Ag, and Au; M′ = Ga, In, Sb, and Bi) with an Ideal Band Gap and High Power Conversion Efficiency. *J. Phys. Chem. C* **2021**, *125*, 21160–21168.

(10) Li, M.; Chen, H.; Ming, S.; Wang, B.; Su, J. First-Principles Calculations of the Structural, Electronic, and Optical Properties of $Cs_2Ag_xNa_{1-x}InBr_6$ Double Perovskites. *Chem. Phys.* **2022**, *559*, 111520.

(11) Volonakis, G.; Haghighirad, A. A.; Milot, R. L.; Sio, W. H.; Filip, M. R.; Wenger, B.;

Johnston, M. B.; Herz, L. M.; Snaith, H. J.; Giustino, F. $Cs_2InAgCl_6$: A New Lead-Free Halide Double Perovskite with Direct Band Gap. *J. Phys. Chem. Lett.* **2017**, *8*, 772–778.

(12) Breternitz, J.; Levcenko, S.; Hempel, H.; Gurieva, G.; Franz, A.; Hoser, A.; Schorr, S. Mechanochemical Synthesis of the Lead-Free Double Perovskite $Cs_2[AgIn]Br_6$ and Its Optical Properties. *J. Phys. Energy* **2019**, *1*, 025003.
